# Advances in cancer immunotherapy: adoptive cell therapy and immune cell engagers in solid tumours

**DOI:** 10.1038/s41416-026-03450-w

**Published:** 2026-04-27

**Authors:** Justin Panasci, Changsu Lawrence Park, Ben Tran, Lillian L. Siu

**Affiliations:** 1 Division of Medical Oncology and Hematology, Princess Margaret Cancer Centre, University Health Network, Toronto, ON Canada; 2 Department of Medical Oncology, Peter MacCallum Cancer Centre, Melbourne, Victoria, Australia

**Keywords:** Cell therapies, Cancer immunotherapy

## Abstract

Adoptive cell therapies (ACT) and immune cell engagers (ICE) redirect or potentiate immune effector function against immune-tolerated antigens expressed on malignant cells, representing a distinct class of engineered immunotherapies beyond immune checkpoint blockade. These strategies have been particularly successful in hematologic malignancies; however, translation to solid tumours has been constrained by antigen heterogeneity, limited immune cell trafficking and persistence, an immunosuppressive tumour microenvironment, and on-target off-tumour toxicity. Despite these barriers, accumulating data and clinical experience with these therapies in solid tumours demonstrate feasibility, scalability, safety, and meaningful clinical activity. In light of recent regulatory approvals of ACT and ICE in solid tumours, we aim to provide a comprehensive clinician-oriented overview of these evolving therapeutic platforms. Herein, we review principles of antigen selection, mechanisms underlying investigational ACT and ICE, current barriers to clinical translation in solid tumours, strategies to overcome these limitations, and future prospects for immune-redirecting drug development in solid tumours.

## Introduction

Distinct from immune checkpoint blockade and conventional targeted agents, adoptive cell therapies (ACT) and immune cell engagers (ICE) are engineered to redirect or potentiate immune effector function against malignant cells. Their modular architectures and broad design flexibility enable diverse mechanisms of action, including direct targeting of surface antigens, recognition of intracellular neoantigens through peptide–MHC complexes, and recruitment of endogenous immune cells into the tumour microenvironment (TME). These properties have accelerated the development of multiple platforms such as tumour-infiltrating lymphocytes (TILs), chimeric antigen receptor (CAR) immune cells, engineered T cell receptor therapies (TCR-T), and multispecific ICE constructs.

The T cell receptor (TCR) is the native complex that recognises presented antigens and initiates T cell activation [1, 2]. This physiological framework provides a useful contrast to CAR and ICE platforms, which redirect T cell activity through engineered modes of antigen engagement. To date, several ACT and ICE agents have achieved regulatory approval, with a growing number of candidates under investigation across solid tumour indications. However, clinical application of these modalities presents challenges, including antigen heterogeneity, limited trafficking and persistence of effector cells, and an immunosuppressive TME that restricts cytotoxic function. Immune-related toxicities such as cytokine release syndrome (CRS), neurotoxicity, and off-tumour effects add further complexity and can vary substantially depending on the targeted antigen and platform.

Although this review focuses on solid tumours, ACT and ICE have achieved their greatest clinical success in hematologic malignancies and autoimmune diseases, as comprehensively summarized by published reviews. CD19-directed CAR T-cell therapies and bispecific T-cell engagers can induce deep, durable remissions in B-cell cancers [3, 4]. More recently, CAR T-cell therapy has been applied in refractory autoimmune diseases by depleting pathogenic B cells [5, 6]. These experiences provide key translational lessons that guide the development of ACT and ICE in solid tumours.

In this review, we provide an overview of design principles, mechanisms of action, current therapeutic applications, and limitations of ACT and ICE in solid tumours. We also discuss barriers to clinical translation, challenges in safe deployment, and emerging strategies aimed at improving specificity, efficacy, and durability of response.

## Target Antigens in Solid Tumours

The human leukocyte antigen (HLA) gene encodes the major histocompatibility complex (MHC) – a series of proteins which are essential for immune modulation, self-tolerance, and foreign antigen recognition [7]. Classical HLA class I proteins (HLA-A, HLA-B and HLA-C) are ubiquitously expressed in nucleated cells and present peptides (pMHC-1), the combination of which serves as antigens for CD8 + T cells to elicit an inflammatory response against non-self antigens. Patient-specific genetic polymorphisms at the HLA locus limit the possible repertoire of pMHC, termed the immunopeptidome [8]. As such, immune surveillance of malignant cells not only depend on the presence of cancer neoantigens, but also on the individual’s immunopeptidome and HLA expression on the tumour/antigen-presenting cells [9]. Indeed, certain HLA haplotypes have been correlated with carcinogenesis and downregulation of tumour HLA expression is a negative predictor of response to immune checkpoint blockade (ICB) [10–12]. ACT and ICE can be engineered to target pMHC on cancer cells that are not recognized by endogenous T cells. Alternatively, cell surface molecules can be targeted directly by CAR without the need for antigen presentation by MHC molecules. This MHC-unrestricted strategy can be advantageous in cancers harboring targetable ancestral antigens (e.g. CD19 and BCMA in lymphoma and myeloma, respectively). Therefore, selecting antigens and mode of targeting in solid tumours is a highly complex exercise that balances patient-specific (immunopeptidome), tumour-specific (antigen expression specificity, intratumoural heterogeneity), and organ-specific (off-tumour on-target toxicity, TME) factors.

### Cancer-Testis Antigens (CTA)

CTAs are highly immunogenic molecules whose expression are normally restricted to the germ cells of the testis in healthy individuals. More than half of CTAs are encoded by the X chromosome, representing approximately 10% of genes on the X chromosome [13]. CTAs are generally intracellular molecules that can be desuppressed in cancer cells to elicit a cytotoxic T lymphocyte response (e.g., PRAME, MAGE-A4 and NY-ESO) [14–16]. Since CTA expression is suppressed by DNA methylation in somatic cells, combination of epigenetic modulators (i.e. 5-azacitadine) with adoptive cell therapy and ICB may optimize CTA overexpression for enhanced targeting [17–19].

### Solid Tumour Enriched Extracellular Antigens

A catalogue of ongoing cell therapy trials in solid tumours and their respective targets has recently been reviewed [20]. Targeted antigens are often drivers of the cancer (e.g., HER2, EGFR) or relatively overexpressed in cancer cells (e.g., Mesothelin, Claudin 18.2). Extracellular antigens can be targeted by both MHC-restricted and unrestricted mechanisms. Wachsmann et al. found that MHC-unrestricted targeting against CD20 was more potent at eliciting an immune response but T cells exhausted more rapidly with increased antigen exposure compared to MHC-restricted targeting [21]. How MHC-restricted and unrestricted targeting compare in solid tumours and may potentially be improved by additional engineering of co-stimulatory domains, remain unknown.

### Identification of novel antigens

Given that there are over 10,000 allelic variants encoded across the six major HLA loci, experimental data for epitope presentation for each variant are inconstant [22]. For example, the HLA-A*02:01 allele has been the most extensively studied and presents the antigens targeted by several FDA approved therapies such as tebentafusp and afamitresgene autoleucel, whereas several other HLA alleles have not been extensively studied [23]. HLA-A*02-01 is amongst the most common HLA class I allele worldwide but its prevalence is variable, ranging from 27% in Europeans to 6.5% in Asians or Pacific Islanders [24]. As such, machine learning algorithms such as NetMHCpan have been employed to predict structures of pMHC-1 neoantigens, which have subsequently been targeted in clinical trials [25–27]. Recent advancements in prediction models such as AlphaFold allow investigators to identify and design targets against predicted tertiary and quaternary structures of pMHC molecules [28]. Johansen et al. recently published a proof of concept of their artificial intelligence pipeline to design binders against pMHC neoantigens [29]. Experimental validation of their predicted binders reported a success rate of ~2%, indicating that this may facilitate the development of novel targets of pMHC where experimental structures are not available, but also highlights the complex biology of antigen presentation that is not yet fully captured by artificial intelligence.

## Mechanisms of Cellular Therapy

### Tumour-infiltrating Lymphocytes (TILs)

**Fig. 1:**
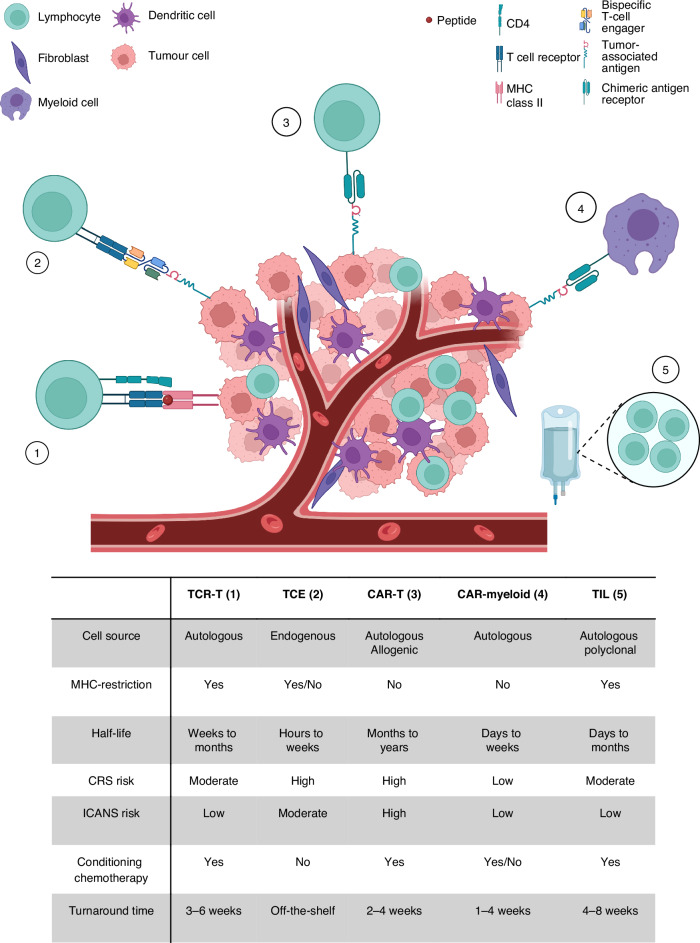
Mechanisms of Cellular Therapy. (1) TCR-T. Autologous T cells expressing tumour-specific T cell receptors recognise intracellular antigens presented as peptide–MHC complexes, leading to T cell activation and tumour cell killing. (2) TCE. Bispecific or multispecific antibodies bind CD3 on endogenous T cells and tumour-associated antigens, promoting immune synapse formation and cytotoxic tumour cell lysis. (3) CAR T. Engineered T cells express chimeric antigen receptors that directly recognise surface tumour antigens in an MHC-independent manner and trigger cytotoxic effector signalling. (4) CAR-myeloid. Myeloid cells engineered with chimeric antigen receptors mediate antitumour activity through phagocytosis, cytokine release, and innate immune signalling. (5) TILs. Polyclonal tumour-resident T cells are expanded ex vivo and reinfused to exploit endogenous antitumour immune repertoires. Key platform characteristics, including cell source, MHC restriction, in vivo persistence, toxicity profiles, conditioning requirements, and manufacturing turnaround time, are summarized in the accompanying table. Abbreviations: TCR-T engineered T cell receptor therapy; MHC major histocompatibility complex; TCE T cell engager; CAR chimeric antigen receptor; TILs tumor infiltrating lymphocytes.

Isolation of TILs from the TME followed by ex vivo expansion and reinfusion showed promising anti-tumour activity in studies performed as early as 1986 [30, 31]. This strategy leverages the patients’ autologous repertoire of TCRs that are presumably recruited by tumour-targeting antigens (Fig. 1). Since then, strategies to selectively expand the likely clones with anti-tumour activity have been developed to reduce off-target toxicity and increase efficacy. Activated CD8+ TILs can be enriched by florescence-activated cell sorting (FACS) using markers such as CD103, CD39, PD-1, and CD137 [32–34]. Furthermore, specific neoantigen targeting can be enriched by co-culture with MHC-matched antigen-presenting cells expressing tumour-informed mutant epitopes. Dual-targeting TILs with transduced CAR against overexpressed tumour antigens prior to expansion have also shown activity in solid tumours [35]. However, TILs are inherently highly variable in their composition and specificity, with unpredictable antigen-affinity and cytotoxicity of expanded clones.

### Engineered T Cell Receptor Therapy (TCR-T)

One strategy to overcome the variability of TILs is to transduce a TCR with known affinity to target neoantigens using the patient’s autologous lymphocytes (Fig. 1). TCR-T therapies remain MHC-restricted and utilize the endogenous inflammatory pathways for anti-tumour effect. Challenges with TCR-T include cross pairing of α- and β-TCR chains from the transduced TCR with those of the native TCR. Such cross-pairing carries the theoretical risk of mixed dimer formation giving rise to new TCRs with unpredictable specificity and toxicity. In addition, there can be competition for accessory signaling proteins between “therapeutic” TCRs and endogenous TCR.

### CAR T cell therapy

CAR T cells are MHC-unrestricted, directly binding to target antigens without the need for antigen-presentation by pMHC complexes (Fig. 1). CARs are composed of an extracellular antigen-binding domain fused with the signaling and co-stimulatory domains of TCR. The extracellular domain is composed of a single-chain variable fragment (scFv), a fusion protein of the variable regions of immunoglobulin heavy and light chains, conferring the specificity of the treatment. The intracellular domains contain the primary CD3 signalling domain and secondary costimulatory domains, with recent approaches incorporating inducible cytokine expression to recruit bystander T cells for immune-mediated anti-tumour effects and clonal amplification [36, 37].

### CAR-expressing innate immune cells

Beyond T cells, CAR technology is increasingly being applied to innate immune populations such as natural killer and myeloid cells. (Fig. 1). CAR-NK cells are the most clinically advanced, combining CAR-mediated specificity with natural cytotoxicity, lower risks of cytokine release syndrome and graft-versus-host disease, and potential for off-the-shelf manufacture [38, 39]. In parallel, myeloid cells (e.g., macrophages and neutrophils) offer complementary advantages, including efficient tumour infiltration, direct cytotoxicity or phagocytosis, and the capacity to remodel the tumour microenvironment through paracrine signaling and antigen presentation [40]. Notably, a first-in-human phase I trial of HER2-targeted CAR-macrophages demonstrated feasibility, tumour infiltration, and preliminary biological activity in patients with HER2-overexpressing solid tumours [41].

### Bispecific and Multispecific Immune Cell Engager Molecules

Immune cell engagers (ICE) comprise a heterogeneous class of anti-cancer therapeutics consisting of multiple scFv domains targeting tumour-specific antigens and immune-cell receptors, thereby serving as a physical bridge enabling orthosteric and allosteric interactions to promote immune activation (Fig. 1). These molecules can be engineered to target multiple tumour antigens to delay antigen escape or target multiple receptors on immune cells to fine tune immune activation and exhaustion of recruited cells [42]. Unlike adoptive cell therapy, ICE have a short half-life and depend on the quality of endogenous immune cells. However, ICE are “off-the-shelf” and can avoid much of the financial and procedural hurdles of adoptive cell therapy.

## Preclinical Models for immune cell engagers and adoptive cell therapy

Unlike cytotoxic chemotherapies that act directly on tumour cells, ACT works indirectly by modulating the patient’s immune system to mount an anti-tumour response. This fundamental difference poses unique challenges in preclinical drug development, prompting the evolution of new experimental models that can support both tumour biology and a functional, human-like immune response. However, these models fail to recapitulate immune-mediated toxicities, which represent an ongoing barrier to clinical translation.

### In vitro and ex vivo models of ACT

Common functional in vitro models involve co-culture of the ACT product and tumour in 2D cell culture systems or 3D organoid models. Efficacy is measured by cytokine secretion by ACT or tumour cytotoxicity as changes in tumour growth kinetics. ex vivo systems utilize patient-derived samples to experimentally manipulate tumours to observe their behavior under controlled conditions. Patient-derived organoids (PDO) have been shown to recapitulate tumour histology and molecular architecture [43]. PDOs can be co-cultured with autologous immune cells from peripheral blood or from the TME to better understand the functional interaction of ACT with the tumour and other immune cells. In such systems, both immune and tumour behavior to cytokine stimulation, checkpoint inhibition and environmental stressors (e.g., hypoxia) can be observed. While both in vitro and ex vivo models are isolated systems that circumvent much of the barriers of ACT in solid tumours (e.g., TME, on-target off-tumour effect and interplay with effector /suppressive immune cells), such assays can give quick readouts to optimize initial cell-therapy design.

### In vivo models of ACT

**Fig. 2:**
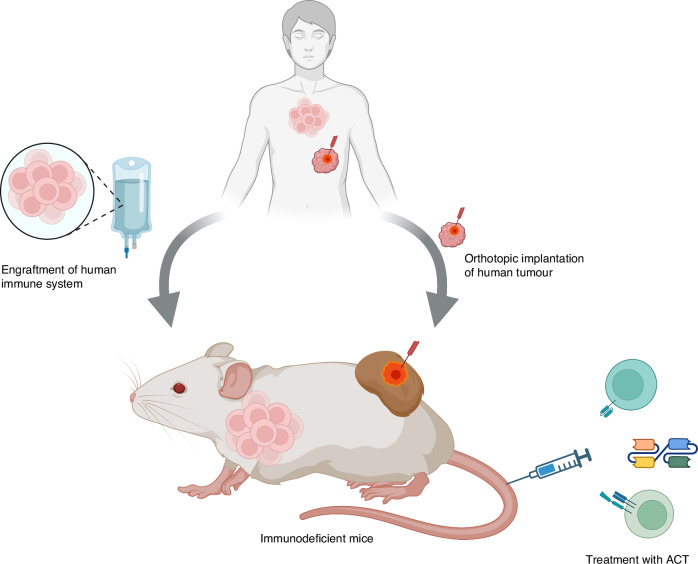
The Humanized Mouse Model. Immunodeficient mice are engrafted with a human immune system and orthotopically implanted with human tumours to generate a semi-autologous tumour–immune context. Following tumour establishment, ACT are administered to assess antitumour activity, immune trafficking, and toxicity in vivo.

Humanized murine models with patient-derived xenograft (PDX) utilize human tumours which are implanted into immunodeficient mice engrafted with human hematopoietic stem cells. Unlike their application in ICB development, ACT requires an HLA-matched or semi-autologous immune system to mitigate allogenic ACT-immune and tumour-immune interactions (Fig. 2). While promising, these models have important limitations: they lack mature lymphoid architecture, have short experimental timeframes, and may generate allogeneic immune responses that confound interpretation.

To improve the translation of novel ACT, ongoing efforts are needed to develop more representative in vivo and ex vivo systems. These should not only simulate the complex interplay between tumour and immune cells, but also capture immune-related toxicities that are increasingly relevant in clinical settings. Advancing these models will be essential for evaluating immunogenicity, pharmacodynamics, and therapeutic windows of next-generation adoptive cell therapeutics. Abbreviations: ACT, adoptive cell therapy.

## Clinical Development

### Early clinical trials

The first generation of ACT and ICE therapies in solid tumours has been defined by a series of early studies focused on safety, feasibility, and biological proof-of-concept (Table 1). TIL therapy marked the earliest autologous approach to demonstrate feasibility beyond melanoma. In a first-in-human phase I study of autologous TILs combined with nivolumab in patients with advanced non-small-cell lung cancer (NSCLC), objective response rate (ORR) was 25%, with several achieving durable disease control despite prior PD-1 therapy failure [44]. Similar studies are ongoing in cervical and head-and-neck cancers (NCT03108495), confirming that tumour-resident lymphocytes can be harvested, expanded, and reinfused safely in epithelial malignancies.

**Table 1:** Status of ACT and TCE Development with Key Targets.

Target	Tumors	TCE	eTCR‑T	CAR‑T	Comments
gp100 (HLA‑A*02:01)	Uveal melanoma	Tebentafusp [50] [APP]	—	—	First approved TCE for solid tumors
		OS benefit vs TPC			
DLL3	ES‑SCLC	Tarlatamab [51] [APP]	—	—	Accelerated FDA approval 2024
		OS benefit vs chemotherapy			
CLDN18.2	Gastric and GEJ cancer	AMG 910 (NCT04260191) [P1]	—	Satricabtagene autoleucel [49] [P2]	Tight-junction molecule overexpressed in gastric tumours
		P1 terminated		PFS benefit	
CLDN6	CLDN6-positive tumours	CTIM-76 (NCT06515613) [P1]		CLDN6 CAR-T [80] [P1]	Proof of concept for combination of CAR T with an amplifying RNA vaccine
		P1 ongoing		ORR 33%	
PSMA	mCRPC	Acapatamab/AMG160 [55][P1]	—	P‑PSMA‑101 (NCT04249947) [P1]	P-PSMA-1 incorporated an inducible caspase 9 safety switch
		Limited by CRS grade ≥ 3 of 16.1% in expansion		grade 5 toxicity (MAS)	
KLK2	mCRPC	Pasritamig [89] [P1]		JNJ-75229414 (NCT05022849) [P1]	Lower response rates and higher ADA titers with SC versus IV administration of Pasritamig
		ORR 8% in heavily pretreated population		P1 ongoing	
EGFRvIII	Glioblastoma	Etevritamab/AMG596 (NCT03296696) [P1]	—	CART‑EGFRvIII [90] [P1]	CAR T limited by antigen heterogeneity and unfavorable TME
		P1 ongoing		CAR‑T infusion decreased intratumoral expression of EGFRvIII	
IL13Rα2	GBM	—	—	IL13Rα2 CAR‑T [91] [P1]	Proof of concept for locoregional delivery in GBM
				DCR 50%	
GPC3	HCC	—	—	CAR‑GPC3 [92] [P1]	Cell-surface antigen highly expressed antigen in HCC
				OS 42% at 1 y; 1 event of grade 5 CRS	
CEA	CEA-expressing tumours	Cibisatamab [93] [P1]	—	CEA CAR‑T [94] [P1]	Obinutuzumab pretreatment reduces ADA
		ORR < 10%		ORR < 10%	
NY‑ESO‑1 (HLA‑A*02:01, *02:05 or *02:06)	Synovial sarcoma; MRCLS	—	Letetresgene autoleucel (NCT03967223) [P2]	—	HLA‑A*02 restricted to target intracellular antigens
			ORR 40%		
MAGE‑A4 (HLA-A*02)	Synovial sarcoma; MRCLS	—	Afamitresgene autoleucel [54] [APP]	—	First engineered TCR‑T approved
			ORR 37%		
KRAS G12D (HLA-C*08:02, HLA-A*11-01, HLA-A*11-02)	KRAS-G12D-mutated tumours	—	NT‑112 (NCT06218914) [P1]	—	Armoured with TGFBR2 knockout to reduce immunosuppression in TME
			P1 ongoing		
KRAS G12V (HLA-A*11-01)	CRC; PDAC; NSCLC	—	NW‑301 V (NCT06478251/06484790) [P1]	—	Impressive efficacy in a heavily pretreated population
			ORR 43%		
GUCY2C	Gastrointestinal tumours	PF-07062119 (NCT04171141) [P1]	—	IM96 (NCT05287165) [P1]	Target involved in intestinal homeostasis and preferentially overexpressed in gastrointestinal tumours
		P1 terminated		ORR 26.3%	

Summary of selected clinical-stage ACT and immune cell engager ICE approaches in solid tumours, organized by target antigen and therapeutic modality, TCE, TCR-T, CAR T. The stage of clinical development for each therapy is indicated as approved (APP), phase 2 (P2), or phase 1 (P1), with key clinical observations and safety considerations listed. References correspond to cited clinical trials. Abbreviations: *ADA* anti-drug antibodies; *CAR T* chimeric antigen receptor T cell therapy; *CRC* colorectal cancer; *CRS* cytokine release syndrome; *DCR* disease control rate; *ES-SCLC* extensive-stage small-cell lung cancer; *GBM* glioblastoma multiforme; *GEJ* gastroesophageal junction; *HCC* hepatocellular carcinoma; *IV* intravenous; *MAS* macrophage activation syndrome; *mCRPC* metastatic castration-resistant prostate cancer; *MRCLS* myxoid/round cell liposarcoma; *ORR* objective response rate; *OS* overall survival; *PDAC* pancreatic ductal adenocarcinoma; *PFS* progression-free survival; *SC* subcutaneous; *SS* synovial sarcoma; *TCE* T cell engager; *TCR-T* engineered T cell receptor therapy; *TME* tumor microenvironment; *TPC* treatment of physician’s choice.

Emerging evidence suggests that PRAME, a CTA presented by HLA-A*02:01, may be an interesting target for TCR-T in solid tumours [14]. For example, IMA203 displayed manageable toxicity and early efficacy in uveal melanoma patients with an ORR of 67% and a median duration of response of 11 months [45]. Beyond CTAs, TCR-T targeting oncogenic driver neoantigens is emerging. Proof-of-concept that *KRAS* mutations can be targeted by T cells in patients comes from a case of *KRAS G12D*–specific T cell transfer achieving regression of metastatic colorectal cancer [46]. Building on this principle, first-in-human *KRAS G12V*-specific TCR-T data were recently presented [47]. Among 14 HLA-A*11:01–positive patients with *KRAS G12V*-mutant pancreatic or colorectal cancer, the ORR was 42.9%, including 50% ORR in higher-dose cohorts, with no grade ≥ 3 CRS and a median PFS ~ 5.8 months. These results confirm the tolerability and reproducible clinical activity of targeting intracellular driver mutations, underscoring the potential of precision adoptive immunotherapy in genomically defined solid tumours.

CAR T cell therapy in solid tumours has produced isolated but instructive successes. The first-in-human IL13Rα2-directed CAR T study in recurrent glioblastoma used intracranial and intraventricular delivery, achieving regression of multifocal disease without dose-limiting toxicity [48]. More recently, CLDN18.2-targeting CAR T cells in advanced gastrointestinal cancers demonstrated an ORR of 35% with predominantly grade 1–2 cytokine-release syndrome in a randomised, open-label, phase II trial [49]. Together, these trials highlight the feasibility of locoregional administration in glioblastoma and the value of targeting lineage-restricted antigens to mitigate off-tumour toxicity.

Immune-cell engager therapies have evolved in parallel. Across these early-phase experiences, common principles have emerged. Safety and manufacturing feasibility have been consistently demonstrated. Biologic readouts, including in vivo persistence, cytokine kinetics, and trafficking, now guide dose selection alongside toxicity. Although response rates remain variable, these foundational studies validate the core platforms and set the stage for subsequent later-phase drug development integrating rational combinations and improved delivery strategies.

### Current FDA approved therapies

The clinical maturation of cellular and engager immunotherapies in solid tumours has culminated in a series of landmark FDA approvals between 2023 and 2025, establishing these modalities as viable treatment classes beyond hematologic malignancies.

Tebentafusp, a gp100–HLA-A*02:01–directed bispecific antibody, provided the first demonstration that an immune engager can improve overall survival in a solid tumour. In the IMCgp100-202 phase III trial for metastatic uveal melanoma, tebentafusp extended median overall survival to 21.7 months versus 16.0 months with investigator’s choice therapy [50]. Toxicities were predominantly manageable cytokine-mediated events and reversible skin reactions.

Tarlatamab, a DLL3 × CD3 BiTE, was approved the same year for previously treated extensive-stage small-cell lung cancer (SCLC) [51]. Lifileucel, an autologous product of unselected, ex vivo-expanded TILs, became the first TIL therapy to receive FDA approval in 2024 for adults with unresectable or metastatic melanoma progressing after standard PD-1 blockade and targeted therapy. In the pivotal phase II C-144-01 study, lifileucel achieved an ORR of 31.4% and median duration of response of 36.5 months [52, 53]. Treatment-emergent toxicities were mainly lymphodepletion-related cytopenias and low-grade cytokine-release events.

Afamitresgene autoleucel (afami-cel), a genetically engineered autologous TCR-T therapy recognizing MAGE-A4 in HLA-A*02:01–positive tumours, received approval in 2024 for metastatic synovial sarcoma. In SPEARHEAD-1, the ORR was 43.9%, median duration 11.6 months, and median overall survival 17.2 months [54]. Toxicities were consistent with cytokine-mediated effects.

Together, tebentafusp, lifileucel, afamitresgene autoleucel and tarlatamab represent some of the first approved ACT and ICE therapies for solid tumours. Their success validates the scalability of individualized cell manufacture, the therapeutic potential of lineage-restricted antigen targeting (MAGE-A4, DLL3), and the clinical feasibility of managing immune-related toxicities. This defines a framework for the next generation of adoptive and bispecific immunotherapies.

### On-target off-tumour effects

Target antigen selection represents a key aspect in drug development of adoptive cell therapy and immune cell engagers. Most antigens overexpressed in solid tumours are shared with healthy tissues. For example, PSMA T cell engager (TCE) acapatamab caused eye disorders (41%), xerostomia (39%) and deafness (5%) in a phase 1 study, presumably related to PSMA expression in certain normal tissues [55]. Strategies to mitigate these on-target off-tumour effects include target antigen saturation in normal tissue and conditional binding using either protease-labile masks, pH-dependent activation and dual-target binding [56]. Combining trastuzumab with HER2 x CD3 T cell engager runimotamab improved tolerability, allowing to reach higher dose levels in a phase I study [57]. TAK-186, an EGFR x CD3 TCE prodrug, is currently being studied in a phase I/II trial in patients with EGFR-expressing solid tumours (NCT04844073). It utilizes peptides that hide key binding domains and require tumour-associated proteases to allow T cell engagement, thereby homing in on the TME. pH-conditional binding of TCE depends on blocking chemicals that are only released from binding sites in the acidic environment characteristic of the TME. This approach was utilized for the preclinical development of a novel EpCAM x CD3 TCE, which shows preferential binding properties at pH 6.0 compared to pH 7.4 [58]. Engineering TCE with binding sites for two distinct tumour antigens can help limit off-tumour toxicity, a design leveraged by the novel construct targeting both cadherin-3 and mesothelin, AMG 305, currently being studied in a phase I trial for advanced solid tumours (NCT05800964).

## Barriers and Challenges in Solid Tumour Applications

### Immunosuppressive tumour microenvironment

Preclinical models consistently demonstrate that the solid TME imposes profound inhibitory pressure on immune effectors (Table 2). Hypoxia is a defining feature, driving T cell exhaustion and metabolic dysfunction. Zhu et al. showed that conventional CAR T cells rapidly lost effector function under hypoxic conditions, with increased expression of exhaustion markers such as PD-1 and TIM-3 [59]. Myeloid-derived suppressor cells and tumour-associated macrophages also impede CAR T cytotoxicity through secretion of TGF-β and IL-10, as well as depletion of arginine and glucose, creating nutrient competition that limits T cell expansion [60]. Together, these findings highlight how metabolic and immunosuppressive pressures in the TME blunt durable activity.

**Table 2:** Barriers to TCE and ACT Efficacy in Solid Tumors Contrasted to Hematologic Malignancies.

Barrier	Solid tumours	Hematologic malignancies	Key references (PMID)
Antigen expression	Heterogeneous expression; antigen loss frequent; many targets intracellular/HLA‑restricted.	Lineage‑restricted antigens with relatively uniform expression (CD19, BCMA).	[95, 96]
Tumour architecture	Dense stroma and abnormal vasculature limiting T‑cell infiltration and distribution.	Circulating and marrow‑resident cells with minimal physical barriers.	[97, 98]
Tumour microenvironment	Immunosuppressive milieu (TGF‑β, adenosine, hypoxia; suppressive myeloid and regulatory cells).	Less compartmentalized suppression; milieu modifiable with lymphodepletion.	[99, 100]
Trafficking	Endothelial dysfunction and chemokine mismatch restricting T‑cell homing.	Direct recirculation through blood and lymphoid tissues.	[101, 102]
Persistence and exhaustion	Chronic stimulation and metabolic stress driving exhaustion; limited durable memory.	Lower tonic signaling; CAR T‑cell persistence achievable after cytoreduction.	[103, 104]
Cell death and antigen release	Hypoxia and necrosis limiting immunogenic cell death and epitope spreading.	Clearance in lymphoid organs supports antigen presentation and secondary priming.	[105, 106]
TCE pharmacokinetics	Poor penetration; antigen sinks; CRS risk heightened with bulky disease.	Predictable exposure to circulating and bone marrow disease with more favourable pharmacokinetics.	[107]
T‑cell starting material	Heavily pre‑treated patients with exhausted/senescent T‑cell compartments.	Earlier‑line use yields fitter starting T cells and more reliable manufacturing.	[108]

Abbreviations: *CAR* chimeric antigen receptor; *CRS* cytokine release syndrome.

### Limited trafficking and persistence

Dense extracellular matrix and abnormal vasculature act as physical barriers to immune infiltration (Table 2). In orthotopic pancreatic and gastric tumour mouse models, CAR T cells accumulated at the tumour periphery but rarely penetrated tumour cores, resulting in heterogeneous killing [61]. Even when trafficking occurs, persistence is often limited. A study of mesothelin-CAR T cells in xenografts showed peak expansion within days, followed by contraction and loss of detectable CAR T within weeks, correlating with tumour relapse [62]. These data reinforce how poor trafficking and short in vivo persistence limit efficacy.

### Antigen heterogeneity and specificity

Preclinical gastrointestinal cancer models illustrate that heterogeneous expression of Claudin-18.2 or mesothelin leads to incomplete tumour clearance, as antigen-negative clones expand under therapeutic pressure. Moreover, several solid tumour antigens are expressed at low levels in normal tissues, creating risks of off-tumour, on-target toxicity. In a phase II trial, low-level expression of Claudin-18.2 in gastric mucosa was sufficient to trigger gastric mucosal lesion or upper gastrointestinal hemorrhage in 14% of patients after CAR T infusion, emphasizing the difficulty of identifying truly tumour-specific targets [49].

### Immune resistance and escape

Adaptive resistance emerges under immune pressure. After adoptive T cell therapy, tumours can evade via alterations in antigen presentation; endogenous T cells are required to restrain antigen-loss escape, highlighting the ease with which tumours adapt to single-antigen pressure [63]. Although IFN-γ is classically immune-activating, it can induce PD-L1 expression in the TME, promoting adaptive immunosuppression and providing a biological rationale for combining immune checkpoint inhibitors with CAR T cell therapies [64].

### Adverse events and dosing limitations

Cytokine release syndrome (CRS) and immune effector cell–associated neurotoxicity syndrome (ICANS) are the dominant toxicities limiting adoptive cell therapy and immune engagers in solid tumours. Importantly, these syndromes are best understood as post-infusion hyperinflammatory states driven by activated immune effector cells rather than strictly dose-dependent toxicities. Their incidence and severity are influenced not only by treatment characteristics but also by patient-specific factors, including tumour burden, baseline inflammatory status and the pre-existing cytokine milieu [65]. CRS, marked by fever, hypotension, and elevated IL-6/TNF-α, occurs in nearly all patients in recent CAR T and T cell engager trials. For example, CLDN18.2 CAR T in gastrointestinal cancers reported CRS in > 95% of patients, though mostly grade 1 and 2 [49]. Similar high incidences are seen with BiTE such as tarlatamab, necessitating step-up dosing and inpatient monitoring [51]. ICANS, though less frequent, remains a critical concern, manifesting as encephalopathy, seizures, or confusion; its unpredictable onset often requires corticosteroid rescue and dose interruption, complicating trial design and limiting dose escalation. High-dose IL-2 given after TIL infusion is associated with capillary leak syndrome (CLS), manifesting as hypotension and vascular permeability, and represents one of the most common toxicities requiring close supportive care in TIL therapy trials [66].

More recently, immune effector cell–associated hemophagocytic lymphohistiocytosis–like syndrome (IEC-HS) has been recognized as a distinct, often life-threatening hyperinflammatory toxicity characterized by cytopenias, hyperferritinemia, coagulopathy and organ dysfunction, typically emerging as CRS is resolving [67]. Case-series data indicate that progressive IEC-HS frequently mandates intensified immunosuppression with high-dose steroids and additional agents such as anakinra and ruxolitinib [68]. This evolving understanding of immune-driven toxicity has informed mitigation strategies across platforms, including step-up dosing schedules for T-cell engagers and the selective use of corticosteroid prophylaxis in higher-risk settings, aimed at reducing toxicity without compromising antitumour efficacy [65]. Together, CLS, CRS, ICANS and IEC-HS narrow therapeutic windows, increase supportive care requirements, and remain major barriers to the broader, safe application of ACT and immune engagers in solid tumours.

### Scalability and cost considerations

Preclinical studies highlight the complexity of ACT manufacturing. Many models require individualized T cell isolation, ex vivo modification, and expansion before infusion, with variability in expansion potential depending on donor source and T cell fitness [69]. This variability foreshadows scalability and cost challenges encountered clinically.

## Strategies to Overcome Barriers

### Enhancing CAR T efficacy

Enhancing CAR T cell efficacy in solid tumours increasingly centers on three engineering pillars. First, armoured CAR T cells that deliver immune-modulating payloads can resist suppression and amplify local immunity. Membrane-tethered interleukin-12 expressed in an antigen-dependent manner augmented interferon gamma programs, remodeled the TME, and improved regional to systemic control in orthotopic ovarian cancer models, without the systemic toxicity seen with soluble cytokine delivery [70]. Similarly, inducible interleukin-18 secretion coupled to a disialoganglioside GD2 chimeric antigen receptor increased effector cytokine production and cytolysis against GD2-positive tumours while retaining antigen-restricted activity in preclinical development, supporting translation to early clinical testing [71]. Beyond cytokine payloads, TGF-β–insensitive CAR T cells armoured with a dominant-negative TGF-β receptor II have shown enhanced proliferation, resistance to exhaustion, and durable tumour control in various preclinical models, leading to an ongoing phase I trial in prostate cancer [72, 73] (NCT03089203).

Second, gene editing can rewire T cells ex vivo or in vivo to improve durability and overcome checkpoint restraint. Multiplex CRISPR-mediated disruption of the T cell receptor alpha constant, beta-2 microglobulin, and PD-1-generated universal EGFR variant III CAR T cells with enhanced activity in glioblastoma models, illustrating how checkpoint deletion and allogeneic manufacturing can be combined [74]. Complementing ex vivo platforms, antibody-targeted enveloped delivery vehicles have been engineered to co-package Cas9 ribonucleoproteins and a CAR transgene. Following systemic administration in humanized mice, these vehicles edited endogenous human T cells in situ and generated gene-edited CAR T cells, thereby advancing the concept of on-body manufacturing and iterative genome engineering [75].

Third, dual and multispecific chimeric antigen receptor designs address heterogeneous antigen expression and reduce single-target escape. In this context, an OR-gate CAR triggers activation when either of two antigens is present, whereas an AND-gate design requires simultaneous engagement of multiple antigens to achieve full signaling output, thereby enhancing specificity [76]. Tandem receptors recognizing folate receptor alpha and mesothelin enhanced killing and limited antigen-negative escape in ovarian cancer models, exemplifying an OR-gate that broadens coverage while maintaining specificity through avidity tuning [77]. Beyond tandem binding, intracellular logic gating can require combinatorial cues for full activation. A logic-gated intracellular network (LINK) architecture that co-opted proximal signaling molecules to build an AND-gate produced selective tumour recognition and robust in vivo tumour control, illustrating a path to discriminate malignant from normal tissues based on multi-antigen context rather than a single epitope [78]. In parallel, CAR vaccine approaches use RNA-based immunization to periodically restimulate adoptively transferred CAR T cells. A nanoparticulate RNA vaccine encoding CLDN6 drove selective expansion and enhanced antitumour efficacy of CLDN6 CAR T cells in solid tumour models [79]. This concept has been translated clinically in the phase 1/2 BNT211-01 trial, which combines CLDN6-directed CAR T cells with a CLDN6-encoding RNA vaccine (CARVac) in CLDN6-positive solid tumours [80]. Together these strategies converge on the same goal, namely increasing potency within the tumour while maintaining control over specificity and toxicity.

### Optimizing immune cell engager therapy

Optimizing immune cell engagers in solid tumours is a multifaceted challenge. Several recent developments suggest paths forward. A key foundation is half-life extension, which improves systemic exposure and reduces continuous administration. For instance, the DLL3 × CD3 engager tarlatamab was engineered with a half-life extension module, and in nonhuman primate models it demonstrated prolonged circulation that supports intermittent dosing regimens while maintaining potent tumour killing [81]. Parallel to that, TME modulation is being integrated directly into engager designs. A novel B7-H3 trispecific killer engager (TriKE) has shown the ability to overcome hypoxia-induced suppression of NK cell activity in head and neck squamous cell carcinoma models, preserving NK cytotoxicity under hypoxic conditions and achieving significant tumour regression [82].

Emerging evidence suggests that dosing schedule and co-stimulation can modulate T cell exhaustion with TCE. Pasritamig KLK2 × CD3 data presented at ESMO 2025 indicated that more extended-interval dosing may preserve TCF1⁺ progenitor-like CD8⁺ cells while limiting accumulation of terminally exhausted subsets. This is consistent with mechanistic work showing that CD3 bispecific antibodies in solid tumours can drive chronic stimulation and exhaustion [83]. Preclinical studies combining a PSMA × CD3 bispecific with 4-1BB co-stimulation demonstrate enhanced T cell proliferation, improved control of larger tumours and induction of memory responses [84], collectively supporting evaluation of extended-interval dosing and rational co-stimulatory combinations to mitigate exhaustion and improve durability of TCE responses.

Finally, investigators are increasingly exploring alternative effector pathways beyond CD3. The B7-H3 TriKE engages NK cells rather than T cells, thereby circumventing T cell exhaustion and potentially reducing cytokine release risks [82]. NK cell–based engagers like TriKEs are gaining traction as complementary or alternative platforms in the solid tumour setting. Together, these innovations, longer half-life designs, rational combinations with immunomodulators, engineered tumour-responsive activation and deployment of non-CD3 effector engagement converge to expand the therapeutic window, deepen infiltration, and enhance durability for immune cell engagers in solid tumours.

## Emerging Technologies and Future Directions

Despite substantial progress in engineering CAR T cells and ICE to overcome the barriers of solid tumours, current platforms remain constrained by manufacturing complexity, limited persistence, and toxicity management. These unmet needs have catalyzed a shift toward next-generation technologies that reimagine how cellular and engager therapies are produced, delivered, and regulated. The following emerging approaches, ranging from allogeneic CAR T platforms to in vivo gene delivery and advanced multispecific engagers, aim to broaden access, improve scalability, and enhance therapeutic durability.

### Allogeneic CAR T

Allogeneic CAR T cells from healthy donors are attractive because they can be manufactured in advance, cryopreserved and delivered as an “off-the-shelf” therapy. Their ex vivo engineering allows genome editing to remove alloreactivity or add functional modules, and the platform enables streamlined development of constructs targeting different antigens or use in rational combinations. Graft-versus-host disease is the key risk of allogeneic CAR T cells and they can be limited in persistence due to rapid elimination by the host immune system [85]. An example of allogeneic CAR T in clinical development is ALLO-316, an HLA-unmatched product targeting both CD-70-positive tumours and alloreactive host T cells in advanced clear cell renal cell carcinoma (NCT04696731). Confirmed responses were observed with single dose in 31% of patients with high tumour CD70 expression, with low incidences of CRS (2% grade 3 + ) and immune effector cell-associated hemophagocytic lymphohistiocytosis-like syndrome (6% grade 3 + ). Robust and sustained CAR T cell expansion occurred due to depletion of CD70-positive host T cells.

### mRNA CAR delivery

Messenger RNA encoding a specific CAR protein can be introduced into autologous T cells via non-viral ex vivo electroporation, producing transient CAR expression without genomic integration. Early studies demonstrate feasibility but limited in vivo persistence due to rapid mRNA degradation, which may require repeated dosing and constrain durable antitumour activity [86]. This transient profile may also reduce prolonged on-target off-tumour toxicity and eliminate risks of insertional mutagenesis. mRNA delivery using lipid nanoparticles or other carriers can additionally enable direct in vivo generation of CAR-expressing immune cells without conventional manufacturing. Thus, mRNA-based CAR strategies represent an active platform for “in-body” programming, although persistence remains the principal current limitation [87].

### Novel bispecific and multispecific engagers

The barriers previously described have shaped the design priorities for newer engager plaftorms [88]. Next generation molecular formats aim to overcome these barriers by improving their pharmacological properties, extending half-life, and enhancing tumour penetration. In addition, by incorporating additional tumour antigens, costimulatory domains, or checkpoint-blocking elements, the antitumour activity of these agents can be optimized by increasing their target specificity, strengthening immune redirection, and mitigating resistance mechanisms.

### Regulatory and logistical considerations

Global alignment is needed for CAR T therapy processes including cell manufacturing, release criteria, and cryopreservation standards. Similarly, bispecific and multispecific antibodies require quality control frameworks that extend beyond those used for conventional monoclonal antibodies. For both therapeutic classes, early recognition and prompt management of toxicity are essential, particularly as these treatments become democratized and increasingly delivered in community practices.

### Combinations with checkpoint inhibitors and other therapeutics

Combinatorial strategies using CAR T cells or bispecific antibodies with immune checkpoint inhibitors have been deployed in clinical trials across multiple solid tumours. The rationale for such combinations includes prevention of T cell exhaustion, overcoming immunosuppressive TME, and enhancing CAR T cell expansion. Other therapeutic partners, such as therapeutic cancer vaccines, mutant-specific inhibitors, and protein degraders may also offer potential synergy, particularly through biomarker-guided patient selection.

## Conclusion

ACT and ICE are beginning to expand the arsenal for treating solid tumours, displaying feasibility, scalability, safety, and meaningful clinical activity. However, broader application is currently limited by antigen heterogeneity, immune suppression within the TME, immune trafficking and limitations in persistence. Next-generation platforms aim to address these constraints while improving precision and pragmatism. Continued progress in antigen discovery, biomarker selection, and preclinical models is required to move the status quo for these therapies in solid tumours. Taken together, ACT and ICE are emerging components of the immunotherapy landscape in solid tumours, with the vision of wider applicability as engineering, safety, and delivery barriers are progressively overcome.

## Data Availability

No new data were generated or analysed in this study.
